# Vacuum-Assisted Abdominal Closure Is Safe and Effective: A Cohort Study in 74 Consecutive Patients

**DOI:** 10.1155/2017/7845963

**Published:** 2017-09-11

**Authors:** R. O. Jensen, T. Buchbjerg, R. M. Simonsen, R. Eckardt, N. Qvist

**Affiliations:** Department of Surgery, OUH, Sdr. Boulevard 29, 5000 Odense C, Denmark

## Abstract

**Background:**

Vacuum-assisted closure (VAC) has, in many instances, become the treatment of choice in patients with abdominal catastrophes. This study describes the use and outcome of ABThera KCI® VAC in the Region Southern Denmark covering a population of approximately 1.202 mill inhabitants.

**Method:**

A prospective multicenter study including all patients treated with VAC during an eleven-month period.

**Results:**

A total of 74 consecutive patients were included. Median age was 64.4 (9–89) years, 64% were men, and median body mass index was 25 (17–42). Duration of VAC treatment was median 4.5 (0–39) days with median 1 (0–16) dressing changes. Seventy per cent of the patients attended the intensive care unit. The 90-day mortality was 15%. A secondary closure of the fascia was obtained in 84% of the surviving patients. Only one patient developed an enteroatmospheric fistula. Patients with secondary closure were less likely to develop large hernias and had better self-evaluated physical health score (*p* < 0,05). No difference in mental health was found.

**Conclusion:**

The abdominal VAC treatment in patients with abdominal catastrophes is safe and with a relative low complication rate. Whether it might be superior to conventional treatment with primary closure when possible has yet to be proven in a randomized study.

## 1. Introduction

Leaving the abdomen open after emergency laparotomy has gained increased popularity during recent decades. Common indications are surgery for abdominal hemorrhage (traumatic/nontraumatic), peritonitis (generalized or local), acute pancreatitis, abdominal compartment syndrome (ACS), fascia dehiscence, and conditions with planned second look [1]. The method inherits a range of problems, such as management of the open abdomen, secondary closure of the fascia, the development of ventral hernias, and enteroatmospheric fistulas. Several different methods and strategies have been argued to minimize the complications and lower nursing requirements. ABThera KCI has been specially developed for abdominal VAC [2].

One of the main outcomes of treatment with open abdomen is mortality, which has been reported to vary between 18 and 65% [3–7] dependent on patient selection. Another important outcome is the secondary closure of the fascia to prevent the development of symptomatic ventral hernias. The rate of obtained secondary fascia closure varies between 48 and 100%, depending on patient selection and treatment indication [2, 7–12]. Finally, the self-evaluated quality of life is important, but seldom reported.

The aim of this study was, prospectively, to evaluate the use of open abdomen with KCI ABThera VAC® in a well-defined geographically region (Region of Southern Denmark) with special focus on indications, course of treatment, secondary fascia closure rate, fistula formation, incisional hernia formation, self-assessed quality of life, and mortality.

## 2. Material and Methods

The study was designed as a prospective cohort study, including all acute hospitals in Region of Southern Denmark with a population of approximately 1.202 mill inhabitants. The patients were included during the period from February 1 to December 31, 2013, at Odense University Hospital, and from April 1 to December 31, 2013, at the district hospitals (Svendborg, Esbjerg, and Aabenraa). During VAC treatment, all patients received antibiotics according to department instructions.

Background data (age, sex, medical history, and BMI) was obtained from the patient's medical record. During treatment, gastrointestinal function (stomach aspiration and bowel movement) was assessed and fluid collected from the VAC system was measured daily. At each dressing change, a bacteria swap should be taken from the fascia edges and the peritoneal cavity for culturing. The outline of the fascia defect was drawn on a translucent sterile dressing placed over the abdomen. Subsequently, the dressing was removed for later calculation of the area of the abdominal wound. In addition, a photo of the defect was taken for documentation before the VAC system was applied. The area from the sterile dressing was later transferred onto a cardboard with known paper density, weighed using a precision paper weight (Jennings JZ 560 scale), and the area of the defect was calculated using the following equation:

(1) $$\begin{array}{c} \text{Paper area}\;\left(\;{cm}^{2}\;\right)=\frac{\text{paper mass}\;\left(g\right)}{\text{paper density}\;\left(g/{cm}^{2}\right)}. \end{array}$$

It was registered whether a complete facial closure was possible and on which postoperative day. The type of closure was registered together with any complications experienced during VAC treatment. Mortality during VAC treatment and within 30 days was registered. The applied negative pressure varied between 25 and 125 mmHg, depending on surgeon choice.

Successful treatment was defined as secondary suture of the fascia after VAC treatment, with no need of mesh. Cases where a second line of VAC treatment was needed were classified unsuccessful together with incomplete secondary closure of the fascia and the use of mesh in connection with secondary closure.

Three months after termination of VAC treatment, all surviving patients were invited to a follow-up. This included a physical examination for any clinical detectable abdominal wall defects, and the patients were asked to fill out a questionnaire to assess their health-related quality of life (SF-36v2®). The answers were analyzed using the authorized scoring software (QualityMetric Health Outcomes TM Scoring Software). Data analysis was performed using the StataIC 12 program and were based on *t*-test (numerical data) and chi^2^-tests (categorical data). A *p* value < 0,05 was considered as statistically significant.

## 3. Results

### 3.1. Indications for Open Abdomen

A total of 74 consecutive patients were included in the study. The patients were divided into 6 different groups according to the indications for open abdomen treatment (Table 1). The most common underlying pathology included 19 patients with intestinal perforations, 12 with anastomotic dehiscence, 10 with ileus, and 6 with ruptured abdominal aortic aneurysm. Abdominal compartment syndrome (ACS) was defined as patients who underwent laparotomy due to abdominal hypertension with onset of manifest multiorgan failure or where primary closure was not possible. In these patients, the underlying pathology was ruptured abdominal aortic aneurism (5), severe intestinal edema following abdominal operation (5), ileus (3), intestinal ischemia (3), and intraperitoneal hemorrhaging (1). Indications for second look were intestinal ischemia, damage control surgery, and inspection of an anastomosis. If a patient had one or more sessions of VAC treatments following an attempt of secondary closure, only the initial was included. The group of others included one patient with a large abdominal wall defect after necrosis of the fascia and one patient with an infected ventral hernia mesh. The last mentioned was the only patient, who developed an enteroatmospheric fistula during VAC treatment. The fistula was located on the ileum.

### 3.2. Procedure Related Data

A total of 70 patients (94,6%) survived until termination of VAC treatment. Successful treatment, defined as complete secondary closure of the fascia without mesh immediately following VAC treatment, was obtained in 84,3% (Table 2). Five patients required a second VAC treatment after the initial closure, and these were included in the unsuccessful group.

### 3.3. Hernia and Quality of Life at Follow-Up

Three months after treatment, 63 of the 74 patients were still alive. And of these, 59 accepted to participate in a follow-up and completed the SF-36® quality of life questionnaire. Clinical evaluation revealed incisional hernia in 10 of the 59 patients (Table 3).

### 3.4. Microbial Results

Of the 74 patients included, results for intra-abdominal bacteria growth analysis were obtained in 63. Thirty-one had only one swap taken, and of these 20 showed bacterial growth. Thirty-two had multiple consecutive swaps taken in connection with dressing changes. Of these, 20 were positive and stayed positive during the treatment period, 4 were negative and stayed negative, 5 went from initial positive to negative, and 3 went from initial negative to positive. The most common bacteria cultured were* Enterococcus faecium*,* Enterococcus faecalis*, and* E. coli* (Table 4).

## 4. Discussion

Successful treatment, defined as achieving complete secondary closure of the fascia, without subsequent repeat of the VAC treatment, was observed in 84,3% of the patients. Risk factors for unsuccessful treatment were being female, having preexisting ventral hernia, prolonged VAC treatment, and large wound area. In trauma patients, secondary closure was obtained in all, whereas ACS patients had the lowest closure rate of 78,6%. At the three-month follow-up, 5 of the 59 patients had developed a symptomatic ventral hernia, which indicated repair. In another 10 patients, a minor ventral hernia had developed. It is not surprising that the development of hernia was significantly higher in the group of patients with an unsuccessful treatment, where all demonstrated a ventral hernia at follow-up. In the two patients with an infected mesh and necrosis of the fascia, the goal of VAC treatment was not to obtain secondary closure of the fascia. The rate of ventral hernia development was comparable to those reported (2–20%) after laparotomy with primary fascia closure [13]. Unfortunately we were not able to get information on the development of ventral hernia in those who did not attend the follow-up. It is possible that additional ventral hernias can present after the three months of our follow-up.

The rate of secondary closure varies greatly in the literature, depending on indications for VAC, technique used, and the underlying disease. Overall, the best results are obtained in trauma patients undergoing damage control surgery with secondary closure rates of up to 100% [8, 9, 11] as obtained in our study. The use of VAC in the treatment of diffuse peritonitis/abdominal sepsis has become more common in recent years. The results are contradictory. In some studies, VAC treatment significantly increases the rate of fascia closure (73% versus 53%) and decreases mortality compared to primary abdominal closure [14]. Others argue that on demand relaparotomy is just as effective as open abdomen with VAC [15]. Our closure rates in peritonitis patients are similar to those previously reported [14, 15], but our mortality of only 10% in these patients is lower than the reported 30% mortality. Patient selection is an important factor in this respect. Other important factors in obtaining fascia closure are duration of VAC treatment and area of the open wound. Also the technique for using VAC may be important [16].

A dreaded complication of open abdomen is the formation of enteroatmospheric fistulas, and the main causes are iatrogenic serosal lacerations, mechanical irritation from adhesive material, and adhesion splitting [17]. The incidence of fistula formation has been reported to vary between 3 and 17% [2, 3]. One patient in our study (1,4%) developed a fistula due to VAC treatment. This is low compared to the rate given in the literature but shows that fistula formation is still a complication to consider [17].

VAC treatment in animal models has been shown to diminish the bacteria load, helping to clear infections [18]. However, the same effect has not been observed in clinical conditions [19], where the development of superinfections may be important [20]. In our study, only 5 patients of 25 with initially positive bacteria growth cleared the intraperitoneal bacteria during VAC treatment irrespective of the current antibiotic treatment. Three patients with an initial sterile culture became infected. The presence of* Enterococcus faecium*,* Enterococcus faecalis*, and* E. coli* indicates contamination from the GI tract, whereas the presence of, for example,* Pseudomonas aeruginosa* indicates nosocomial infection. A tailored antibiotic treatment according to the results of cultures from abdominal fluid might have a potential to improve patient treatment. Further prospective studies are needed to solve the problem.

Another important outcome is the self-evaluated quality of life. Patients with unsuccessful treatment rated their physical health significantly lower than patients in the successful group, which was to be expected. Besides being statistically significant, the difference was also of clinical relevance. Studies have shown that patients treated with open abdomen, who could achieve closure of the fascia, had the same quality of life as other patients undergoing similar surgery [5] or as the general population [21], whereas patients with an incisional hernia scored lower [7, 21, 22]. The latter result was confirmed in this study. However, these findings are subjected to confounders, such as comorbidity and the presence of stomas, which will affect the physical quality of life. The relative low number of patients in the present study did not allow sufficient statistical analysis on this problem. The difference in physical health had no influence on the mental health. This could be explained by the fact that the patient despite their physical disabilities just was grateful for having survived a serious and life-threatening disease or condition. Mortality after three months was 15%, highest in the ACS group. Complications to VAC treatment were not observed in any of the mortality cases.

The multicenter design involving several different physicians in different medical centers is a limitation in our study, since proper technique using the open abdomen VAC materials is essential to treatment success [16]. It appears that the indication for open abdomen treatment is decisive for outcome. However, the results from our study showed that the abdominal VAC treatment, in the treatment of patients with abdominal catastrophes, is safe with a relative low complication rate. Whether it might be superior to conventional treatment with primary closure when possible has yet to be proven in a randomized study.

## Figures and Tables

**Table 1:** Patient background data and data regarding VAC treatments. Unless otherwise stated, the results are given as median (range) or number *N* (%).

	All	Secondary peritonitis	Abdominal compartment syndrome (ACS)	Fascia dehiscence	Planned second look	Trauma	Others
*N*	74	29	17	11	10	5	2
Age (years)	64.4 (9–89)	63 (9–85)	71 (23–80)	69 (53–85)	63 (54–89)	28 (20–60)	61.5 (58–65)
Sex, men	47 (64%)	19 (65,52%)	12 (70,59%)	5 (45,45%)	6 (60%)	5 (100%)	0 (0%)
BMI	25.2 (16.90–41.81)	24.94 (16.90–34.97)	24.01 (19.38–32.10)	25.6 (21.56–39.00)	25.94 (20.90–41.81)	27.62 (19.98–30.25)	24.3 (17.68–30.92)
Duration of VAC (days)	4.5 (0–39)	4 (2–18)	5 (3–13)	6 (2–39)	2 (1–10)	6 (0–10)	3 (2–4)
VAC-changes	1 (0–16)	1 (0–7)	1 (0–6)	1 (0–16)	0 (0–3)	1 (0–4)	0,5 (0-1)
Intensive care unit	52 (70%)	21 (72.41%)	14 (82.35%)	3 (27.27)	8 (80%)	5 (100%)	1 (50%)
Largest laparotomy area measured (cm^2^)	143.5 (27–473)	203 (57–500)	140 (87–350)	116 (27–496)	121.5 (43–326)	263.5 (126–406)	173 (106–240)
Largest fluid output per day (ml)	1000 (0–11000)	1000 (0–3200)	1125 (500–11000)	900 (500–2300)	1000 (700–1900)	800 (500–4000)	500 (500-500)
Treatment success	59 (84%)	23 (82.14%)	11 (78.57%)	10 (90.91%)	10 (100%)	5 (100%)	0 (0%)
Died prior to closure	4 (5%)	1 (3.45%)	3 (17.65%)	0 (0%)	0 (0%)	0 (0%)	0 (0%)
Died within 3 months	11 (15%)	3 (10.34%)	6 (35.29%)	1 (9.09%)	1 (10%)	0 (0%)	0 (0%)

**Table 2:** Clinical background data and procedure related data in those with an unsuccessful and successful treatment. Unless otherwise stated, the results are given as median (range) or number *N* (%).

	Treatment unsuccessful	Treatment successful	*p* value
*N* (%)	11 (15.7%)	59 (84.3%)	
Age (years)	64.3 (20–79)	61.5 (9–89)	0.62
Sex, men *N*	4 (36.4%)	41 (69.5%)	0.035
BMI	25.4 (17.68–34.97)	26.2 (16.90–41.81)	0.68
Known ventral hernia prior to treatment	3 (27.3%)	4 (6.8%)	0.036
Duration of VAC (days)	10.8 (2–39)	5.2 (0–15)	0.0013
VAC-changes	4.2 (0–16)	1.6 (0–6)	0.0012
Wound area (cm^2^)	250 (57–496)	157.1 (27–500)	0.0059
Fluid output max (ml/day)	1224 (500–3100)	1190 (0–11000)	0.89
Died within 3 months	4 (36.4%)	3 (5.2%)	0.002

**Table 3:** Results from the three-month follow-up clinical examination and the quality of life questionnaire. The results are given as number *N* (%). For the SF-36, the results given are mean value and confidence interval (95% CI).

	Treatment successful	Treatment unsuccessful	*p* value
Total	52 (88.1%)	7 (11.9%)	
Ventral hernia	6 (11.5%)	4 (57.1%)	0.002
Large hernia needing repair/already repaired	2 (3.8%)	3 (42.6%)	0.002
SF-36 physical (95% CI)	41.75 (26–37)	31 (22–63)	0.0057
SF-36 mental (95% CI)	45.8 (21–69)	40.5 (21–70)	0.3193

**Table 4:** The results of microbial culturing from peritoneal fluid during VAC changes.

Microbe	Number of patients
*Enterococcus faecium*	29
*Enterococcus faecalis*	11
*Escherichia coli*	10
Yeasts	6
*Pseudomonas aeruginosa*	5
*Enterobacter cloacae*	5
*Proteus vulgaris*	3
*Staphylococcus haemolyticus*	3
*Klebsiella oxytoca*	2
*Klebsiella pneumoniae*	2
*Morganella morganii*	2
*Stenotrophomonas maltophilia*	2
*Aeromonas* species	1
*Citrobacter youngae*	1
*Serratia marcescens*	1
*Staphylococcus aureus*	1
*Bacillus cereus*	1
